# Association of ATG5 gene polymorphism with Parkinson’s disease in a Han Chinese population

**DOI:** 10.1007/s13760-021-01814-y

**Published:** 2021-10-18

**Authors:** Jing Han, Ganghua Feng, Jibao Wu, Yi Zhang, Zhipeng Long, Xiaoxi Yao

**Affiliations:** 1 School of Basic Medical Sciences, Xiangnan University, Chenzhou, 423000 China; 2 Department of Neurology, Chenzhou First People’s Hospital, Chenzhou, 423000 China

**Keywords:** Autophagy, Parkinson’s disease, ATG5, Single-nucleotide polymorphisms

## Abstract

**Purpose:**

There is growing evidence that autophagy-related gene 5 (ATG5) is involved in neural development, neuronal differentiation, and neurodegenerative diseases. The purpose of this study was to investigate the association between ATG5 gene single-nucleotide polymorphisms (SNPs) and Parkinson’s disease (PD) in the Han population.

**Methods:**

A case–control study was conducted in 120 PD patients and 100 healthy volunteers. MassArray platform was used to analyze polymorphisms in three different regions of ATG5 gene (rs510432, rs573775 and rs17587319). In the included subjects, 50 PD patients and 50 healthy volunteers were selected, and the plasma ATG5 concentration was detected by enzyme-linked immunosorbent assay (ELISA). The allele and genotype frequencies of SNPs were assessed using the SHEsis program.

**Results:**

We found a significant correlation between rs17587319 and PD, and the subcomponent showed a high correlation between rs17587319 with cognitive impairment and age at onset in PD patients. At the same time, the total plasma ATG5 level of PD patients and the plasma ATG5 expression level of early-onset Parkinson’s disease (EOPD) patients were significantly higher than the control group, while there was no significant difference of ATG5 expression between late-onset Parkinson’s disease (LOPD) patients and the control group.

**Conclusion:**

These findings suggest that genetic variations in the ATG5 gene and low levels of the ATG5 protein are associated with susceptibility to PD and with cognitive impairment in PD patients. ATG5 could be a potential biomarker to assess the severity and prognosis of PD.

## Introduction

Parkinson’s disease (PD) is a common clinical neurodegenerative disease, which often occurs in elderly people. The main pathological features of PD are the degeneration and progressive loss of dopaminergic neurons in the substantia nigra dense part of the basal ganglia [1]. Some studies showed that the incidence of PD was increasing year by year [2, 3]. The risk factors of PD mainly include age, gender and some environmental factors [4]. For most patients, the cause of the disease is unknown. Only a small proportion of patients (about 10%) have been identified with different genetic factors for pathogenicity. The main motor symptoms of PD are static tremor, muscular rigidity, bradykinesia, and postural disorders [5]. A large amount of evidence supports that α-SYN is a key factor in the initiation and progression of neurodegeneration in PD [6, 7]. Some recent researches proved that genetic alterations could cause or affect the susceptibility of PD [8]. However, the genetic factors of PD remain largely unknown.

A large body of evidence suggests that autophagy deficiency is central to the etiology and pathogenesis of PD. Many genetic mutations that regulate autophagy genes or are associated with the autophagy lysosomal pathway (ALP) have been identified as risk factors of PD [9]. ALP is one of the main mechanisms for cell repair or elimination of abnormal proteins. It consists of four stages: induction of autophagy, formation of autophagosomes, fusion with lysosomes, and disintegration of autophagosomes. The damage to any of the links may lead to the aggregation of abnormal proteins and cell death [10]. Autophagy disorders have been found to be associated with the occurrence and development of neurodegenerative diseases [11–13]. For PD, abnormality of ALP could affect α-SYN aggregation and are closely associated with the risk of PD [14, 15]. However, the reports of the association between genetic mutations of autophagy-related genes (ATG) and risk factors of PD are lacking.

Autophagy is carried out through the activity of multiple ATG. Up to now, 31 ATG genes have been identified [16]. These genes play important roles in different stages of autophagy. Abnormal expression of any gene can lead to autophagy defect and induce a series of pathological changes. ATG5 plays a key role in the formation of autophagosomes [17]. ATG5 and ATG12 can form a complex by covalent bond. The complex exists in the form of a single protein regardless of whether autophagy activity is initiated. ATG5 plays an important role in regulating the direction of autophagosome membrane bending during the occurrence and development of autophagy [18]. In recent years, it has been found that the abnormal expression or gene deletion of ATG5 is closely related to the occurrence of a variety of neurodegenerative diseases [19, 20]. In cerebral palsy, some researchers demonstrated an association of an ATG5 gene variant and low level of ATG5 protein with cerebral palsy and stronger associations with severe clinical manifestations were identified [13]. 2 single-nucleotide polymorphisms (SNPs) in ATG5 have also been found they were associated with the susceptibility to epilepsy [21]. In neurons, mice with neuron-specific ATG5 knockout showed progressive motor loss. Neuronal cells of ATG5 knockout mice could accumulate inclusionary bodies and death [22]. In addition, a functional variant of ATG5 in sporadic PD has also been found [23]. These data suggest that the expression and gene polymorphism of ATG5 can affect the development of PD.

In summary, we analyzed three single SNPs from different regions of the ATG5 gene in 120 patients with PD and 100 controls. Furthermore, we used a plasma ATG5 protein assay to clarify the functional association of ATG5 polymorphisms and PD. We aimed to investigate the association of 3 ATG5 (rs510432, rs573775 and rs17587319) variants with the susceptibility of PD among the Han Chinese population.

## Materials and methods

### Subjects

A case–control study was conducted. The control group (*n* = 100) consisted of volunteers who had no family history of PD, symptoms of other neurodegenerative diseases, or similar diseases; The PD group (*n* = 120) consisted of PD patients who were diagnosed with PD according to the UK Brain Bank diagnostic criteria and had no symptoms of other neurodegenerative diseases or a family history of similar diseases. All of them were Han nationality, and there was no significant difference between age and sex.

The age of onset of PD was defined as the time when a patient first noticed symptoms of PD. PD onset before 50 years old was defined as early-onset Parkinson’s disease (EOPD), otherwise, it is considered late-onset Parkinson’s disease (LOPD); According to the Chinese Montreal Cognitive Assessment (MoCA), PD patients were divided into cognitively normal group (≥ 26 points) and cognitively impaired group (< 26 points) [24]. According to movement disorders, there are three subtypes: postural and gait disorders, tremor disorders and intermediate disorders. Clinical data of all subjects were collected. This study was approved by the Ethics Committee of Chenzhou First People’s Hospital. All sample collection and data investigation were approved by informed consent and signature of patients and their families, which was in accordance with the Helsinki Declaration of the World Medical Association.

### Sample collection and genotyping

Venous blood was collected by EDTA tube and centrifuged at 4 ℃ for 1500 g for 20 min. Plasma was separated and stored at − 80 ℃.Genomic DNA was extracted from peripheral venous blood of subjects using whole blood DNA extraction kit (BioTeke. Beijing, China).

SNPs were genotyping using Sequenom MassARRAY (Sequenom, San Diego, USA). The Sequenom MassARRAY system was designed in five steps, including template amplification, dephosphorylation, single base extension (SBE) reaction, sample pretreatment, transfer and genotyping, and bioinformatics. The person who analyzed genotypic results are blind to the subjects’ clinical data. Multiplex PCR primer sequences (Sangon Biotech, Shanghai, China) for these SNPs were listed in Table 1.

**Table 1:** All primer sequences used in polymerase chain reaction

SNPs	Primer sequences
rs510432	
Sense sequence	5′-GGGGCAGTACGCTTGAACT -3′
Anti-sense sequence	5′-TTGGATGGGTGGGAGGGTTC -3′
rs573775	
Sense sequence	5′-TGTCCTTATGCCATACCT-3′
Anti-sense sequence	5′ - TTCAAATCCCTACTCTGC-3′
rs17587319	
Sense sequence	5′-TTCAAATCCCTACTCTGC-3′
Anti-sense sequence	5′-TATCACAAATAAAATCTT-3′

*SNPs* single-nucleotide polymorphisms

### Selection of SNP sites

Combined with references[13, 23], the SNPs of ATG5 gene with locus gene frequency greater than 0.05 in Han population were selected from the dbSNP database (http://www.ncbi.nlm.nih.gov/SNP): rs510432, rs573775 and rs17587319. Rs510432 SNP is located in the upstream region of the ATG5, while the other two SNPs are located in introns.

### Plasma ATG5 levels

To perform the enzyme-linked immunosorbent assay (ELISA) test, the frozen sample is first taken out of the refrigerator and brought to room temperature. Plasma levels of ATG5 in 50 patients and 50 control cases were measured using a human ATG5 ELISA assay kit (Cloud Clone Corp. Houston, USA) according to the manufacturer’s protocol. The absorbance at 405 nm was measured using a SpectraMax 190 Microplate spectrophotometer (Molecular Devices. California, USA). ATG5 levels in plasma were normalized to kit standards, and the data were expressed as (MEAN ± Standard Deviation, MEAN ± SD).

### Statistical analysis

The following analyses were performed on the SHEsis online software platform (http://analysis.bio-x.cn), including comparison of allele and genotype frequencies, Hardy–Weinberg equilibrium test (HWE), haplotype association analysis, and calculation of paired linkage disequilibrium. Student *t* test was used for analysis of protein levels. For the result statistics of counting data, we carried out Chi square test. Statistical analysis was performed using SPSS (version 21.0) and PRISM (version 6.0). *P* < 0.05 was recognized as statistical difference.

## Results

### Comparison of baseline data between PD group and control group

There was no statistical significance in gender, age, Body Mass Index (BMI) and education level between PD group and control group (*P* > 0.05), as shown in Table 2. These data show that the two groups are comparable.

**Table 2:** Baseline data of PD patients and control group (MEAN ± SD)

Project	Control (*n* = 100)	PD (*n* = 120)	*P*
Gender (male/female)	58/42	66/54	0.655
Age	62.62 ± 10.11	62.11 ± 11.23	0.723
BMI (kg/m^2^)	23.13 ± 2.43	23.16 ± 2.34	0.919
Years of education (y)	10.83 ± 3.50	10.05 ± 3.54	0.105

### The allele and genotype frequency of SNPs locus of ATG5 gene

The genotype distribution of ATG5 polymorphism loci in PD and control group was in line with HWE, which was representative of the population. We then analyzed the association between the cases and the control group, only the allele frequency and genotype distribution of rs17587319 were significantly different between the control group and the PD group (*P*_Allele_ < 0.0001, *P*_Genotype_ < 0.0001), and the frequencies of C allele and CC genotype were significantly higher in PD patients. These results indicate that individuals with rs17587319 CC genotype have a significantly higher risk of developing PD than individuals with GG genotype. As shown in Table 3.

**Table 3:** Allele frequency and genotype distribution of SNPs loci

rs510432	Allele		*P*	Genotype			*P*	HWE
	A	G		AA	AG	GG		
Control	82 (0.410)	118 (0.590)	0.831	20 (0.200)	42 (0.420)	38 (0.380)	0.949	0.187
PD	96 (0.400)	144 (0.600)		22 (0.183)	52 (0.433)	46 (0.383)		0.287
rs573775	C	T		CC	CT	TT		
Control	104 (0.520)	96 (0.480)	0.328	26 (0.260)	52 (0.520)	22 (0.220)	0.223	0.677
PD	136 (0.567)	104 (0.433)		32 (0.267)	72 (0.600)	16 (0.133)		0.015
rs17587319	C	G		CC	CG	GG		
Control	144 (0.720)	56 (0.280)	** < 0.0001**	52 (0.520)	40 (0.400)	8 (0.080)	** < 0.0001**	0.937
PD	216 (0.900)	24 (0.100)		100 (0.833)	16 (0.133)	4 (0.033)		0.005

After Bonferroni’s correction, *p* less than 0.017 was considered statistically significant

*PD* Parkinson’s disease, *HWE* Hardy–Weinberg equilibrium test

The significant *P* values are indicated in bold

### Linkage disequilibrium of SNP locus of ATG5 gene and association analysis of haploid and PD

Linkage disequilibrium analysis was conducted on the three SNP sites of ATG5, and the results showed that D ‘among the three was all less than 0.8, as shown in Table 4. To determine whether the SNPs would have greater predictive value when analyzed together, haploid analysis was performed on the above 3 sites (haplotype with frequencies less than 3% were ignored), and a total of 7 haplotypes were obtained, among which 6 haplotype showed statistically significant differences in frequency distribution between the PD group and the control group. The frequency of CAC and TGC haplotype in the case group was significantly higher than that in the control group, so there is a risk for PD. As shown in Table 5.

**Table 4:** The linkage disequilibrium among the SNPs in ATG5

D’/r^2^	rs510432	rs17587319
rs573775	0.443/0.111	0.066/0.001
rs510432	–	0.018/0.000

**Table 5:** Analysis of haplotype in ATG5

Haplotype	Case (freq)	Control (freq)	Pearson’s p	Odds Ratio [95%CI]
C A C	79.84 (0.333)	20.03 (0.100)	** < 0.0001**	4.437 [2.601–7.571]
C A G	16.16 (0.067)	14.74 (0.074)	0.776	0.899 [0.432–1.870]
C G C	38.10 (0.159)	54.97 (0.275)	**0.003**	0.492 [0.309–0.784]
C G G	1.90 (0.008)	14.26 (0.071)	**0.0004**	0.103 [0.022–0.474]
T A C	0.00 (0.000)	45.53 (0.228)	** < 0.0001**	−
T G C	98.06 (0.409)	23.47 (0.117)	** < 0.0001**	5.145 [3.115–8.498]
T G G	5.94 (0.025)	25.29 (0.126)	** < 0.0001**	0.174 [0.070–0.434]
Global result			** < 0.0001**	

The significant *p* values are indicated in bold

### Analysis of the relationship between SNP of ATG5 gene rs17587319 and cognitive impairment in PD patients

PD is a multifactorial and complex disease with a variety of clinical and pathological features. Therefore, subgroup analysis was conducted according to different criteria. We found that there were significant differences in allele frequencies and genotypes of rs17587319 between cognitive impairment and normal PD groups (*P*_Allele_ = 0.003, *P*_Genotype_ = 0.013), and the frequencies of C allele and CC genotype were significantly higher in PD patients with cognitive impairment. As shown in Table 6.

**Table 6:** Comparison of allele frequency and genotype distribution of rs17587319 locus between cognitive impairment group and normal PD patients

Group	rs17587319						*P*
	C	G	*P*	CC	CG	GG	
Cognitive impairment	112 (0.966)	4 (0.034)	**0.003**	55 (0.948)	2 (0.034)	1 (0.017)	**0.013**
Normal group	106 (0.855)	18 (0.145)		47 (0.758)	12 (0.194)	3 (0.048)	

The significant *P* values are indicated in bold

### Relationship between SNP at ATG5 rs17587319 and clinical parameters in PD patients

Then group analysis is performed as shown in the table below. The results show that SNP at rs17587319 was correlated with the onset age of PD patients (*P*_Allele_ < 0.0001, *P*_Genotype_ = 0.00024), and had nothing to do with gender, PD subtype and first limb (*P* > 0.05). As shown in Table 7.

**Table 7:** Relationship between SNP at rs17587319 and clinical parameters in PD patients

Clinical parameters	rs17587319		*P*	rs17587319			*P*
	C	G		CC	CG	GG	
Age							
EOPD	22 (0.688)	10 (0.312)	** < 0.0001**	9 (0.562)	4 (0.250)	3 (0.188)	**0.00024**
LOPD	194 (0.933)	14 (0.067)		91 (0.875)	12 (0.115)	1 (0.010)	
Gender							
Male	119 (0.902)	13 (0.098)	0.931	55 (0.833)	9 (0.136)	2 (0.030)	0.975
Female	97 (0.898)	11 (0.102)		45 (0.833)	7 (0.130)	2 (0.037)	
PD subtype							
Postural gait disorder	96 (0.897)	11 (0.103)	0.917	42 (0.824)	7 (0.137)	2 (0.039)	0.997
Tremor type	71 (0.910)	7 (0.090)		33 (0.846)	5 (0.128)	1 (0.026)	
Middle type	54 (0.915)	5 (0.085)		25 (0.833)	4 (0.133)	1 (0.034)	
First limb							
Left	92 (0.902)	10 (0.098)	0.931	43 (0.843)	6 (0.118)	2 (0.039)	0.875
Right	124 (0.899)	14 (0.101)		57 (0.826)	10 (0.145)	2 (0.029)	

*EOPD* early-onset Parkinson’s disease, *LOPD* late-onset Parkinson’s disease, *PD* Parkinson’s disease, The significant *p* values are indicated in bold

### Assay of plasma ATG5

**Fig. 1:**
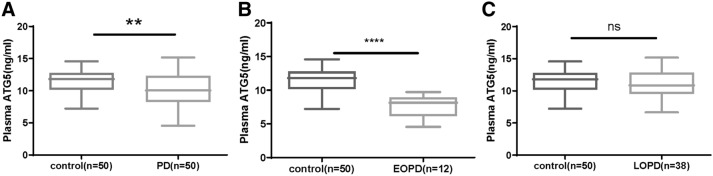
Plasma ATG5 concentration in controls and PD patients. **A** Expression of ATG5 in plasma of 50 controls and 50 PD patients, (**B**) Expression of ATG5 in plasma of 50 controls and 12 EOPD patients, (**C**) Expression of ATG5 in plasma of 50 controls and 38 LOPD patients. *P < 0.05, ns denotes no statistical significance

To further clarify the difference in plasma ATG5 concentration between PD patients and the control group, ELISA was used to measure plasma samples collected, and the results showed that the expression of ATG5 in plasma of PD patients was significantly lower than that of the control group (Fig. 1A,  *P*= 0.0063). Further analysis showed that there was a significant difference in plasma ATG5 expression between EOPD patients and the control group (Fig. 1B, *P*< 0.0001), there was no significant difference in ATG5 expression between LOPD patients and the control group (Fig. 1C, *P*>.05).

## Discussion

Autophagy is a process in which cells degrade damaged organelles and some macromolecules by using their own lysosomes. Autophagy can be induced when eukaryotic cells are stimulated by external conditions such as hypoxia and insufficient nutrition or when cells themselves are damaged or cytoplasmic aggregation [25, 26]. It is well known that autophagy disorders and lysosomal damage are present in a wide range of neurodegenerative diseases, and neurons are particularly susceptible to lysosomal dysfunction. This is because more than two thirds of lysosomal storage diseases manifest as central nervous system dysfunction [27]. In recent years, some ATGs have been found to perform an increasingly important role in PD. Some germline and somatic mutations of ATGs were also linked to PD [28, 29]. Yet the specific correlation between ATG5 and PD susceptibility remains unclear.

In the current study, we first linked a genetic variant within the ATG5 gene to PD. The allele frequency and genotype frequency of the rs17587319 in ATG5 showed a strong correlation with PD patients. In addition, subcomponent showed a high correlation between rs17587319 with cognitive impairment and age at onset in PD patients. These results suggest ATG5 play a more important role in EOPD patients. At the same time, the ATG5 level of plasma in PD patients and EOPD patients were significantly higher than the control group, while there was no significant difference of ATG5 expression between LOPD patients and the control group. These results indicate that the variant of ATG5 may affect the occurrence of autophagy in PD patients by changing the expression of ATG5 protein.

In the studies on the association between ATGs and neurodegenerative diseases, the SNPs and abnormal protein expression of ATGs are considered to be closely related to the development of a variety of diseases. Knockout of the core autophagy protein, ATG5 or ATG7, in mouse neurons caused accumulation of polyubiquitinated inclusion bodies and behavioral defects [22, 30]. A recent genome-wide screening in the striatum has suggested that many ATGs were associated with Huntington’s Disease [31]. Some studies have found that motor neuron-specific ATG7 knockout mice carrying the pathogenic mutation of SOD1 may induce Amyotrophic Lateral Sclerosis [32]. The gene variant and abnormal expression of ATG5 and ATG7 were also existed strong association with cerebral palsy patients [13, 33]. The effect of ATGs on PD is mainly through affecting the formation of α-Syn inclusion bodies. In motor dysfunction mice, ATG7 knockout promotes the formation of α-SYN inclusion bodies of dopamine neurons [34]. In addition, α-SYN inclusions have been shown to reduce omega formation by inducing ATG9a mislocalization [35, 36]. The results of our study further indicate that SNP and expression of ATGs are closely related to the occurrence of neurodegenerative diseases including PD.

ATG5 rs510432 SNP have been shown to influence certain diseases of the immune system, cancer and neurodegenerative diseases. Rs510432 could increase promotor activity of ATG5 and was associated with childhood asthma [37]. Among lung adenocarcinoma patients with EGFR-mutant, ATG5 rs510432 have been proved to contribute to disease prognosis [38]. For neurodegenerative diseases, the genotypes of over-dominant in rs510432 between controls and epilepsy also showed significant difference [21]. In addition, rs573775T* allele was regarded as a risk factor for lupus erythematosus in carriers of the high IL-10 producer genotype. ATG5 rs573775 T* allele seems to influence lupus erythematosus susceptibility, cytokine production and disease features [39]. In hepatocellular carcinoma and epilepsy, rs573775 was not associated with the susceptibility to these diseases [21, 40]. In our study, allele frequency and genotype distribution of rs510432 and rs573775 was no significantly different between the control group and the PD group. Rs17587319 of ATG5 has only been studied in asthmatic patients. The results showed no significant association of rs17587319 with increased asthma risk [41]. Our researches showed rs17587219 of ATG5 showed a strong correlation with susceptibility to PD.

Subgroup analysis based on clinical classification plays an important role in the study of the association between SNPs and PD. Subgroup analysis can identify the association between SNPs and disease subtypes and reduce the influence of phenotypic heterogeneity on the study. The variant allele T of rs3904099 in TLR2 was associated with the susceptibility of LOPD patients [41]. Rs3025039 of VEGF existed significant association with male PD patients and LOPD patients [42]. The subgroup analysis of our results showed a high correlation between rs17587319 with cognitive impairment PD patients and EOPD patients. However, it is worth noting that the C allele and CC genotype of rs17587319 are risk factors for PD, but the frequency of these alleles and genotypes was higher in LOPD patients in subtype analysis. For this phenomenon, we believe that the possible reasons are as follows: First, the limited number of samples collected in this study may affect the accuracy of the results to some extent. Second, there is no literature suggesting that the severity of PD patients can be determined by the time of first onset. For example, Claudia Feitsa-Santana et.al believed that color vision impairment in EOPD patients was more serious than that in LOPD [43]. However, some researchers believe that although LOPD patients show a shorter course of disease, their cognitive abilities, including executive function and visuospatial function, are more severely impaired than EOPD patients [44]. Differences in these results may be related to individual differences among the patients enrolled by the investigator.

Except for ATG5 gene variants, the expression of plasma ATG5 could also characterize susceptibility to neurodegenerative diseases. In the study about susceptibility of Alzheimer’s disease, plasma levels of ATG5 were significantly elevated in patients with dementia or mild cognitive impairment compared with the control participants [45]. Beyond that, lower plasma ATG5 levels were found in child cerebral palsy patients than control participants [13]. The ATG5 level of plasma in PD patients and EOPD patients were significantly higher than the control group in our study. These findings suggest the expression of ATG5 protein might be regulated by variation of ATG5 and activates ALP to affect the development of PD. While Chen et.al described, ATG5 gene expression level in the PD patient was significantly elevated than that in controls which is contrary to our conclusions [23]. This may be because Chen et al. used qRT-PCR to detect the expression of ATG5 mRNA in the leukocytes of patients, which was different from the detection methods and subjects in this study.

In conclusion, the present study first explored the association between variants of ATG5 and susceptibility of CP patients. We found a significant correlation between rs17587319 and PD. A high correlation between rs17587319 with cognitive impairment PD patients and EOPD patients has also been found. In addition, the low expression of plasma ATG5 was significantly associated with PD and EOPD. However, this study also has some limitations. First of all, the total sample size included in this study is not enough, which may cause our results to ignore some weak correlations. Second, more rare SNPs need to be found and analyze the association with PD. In the future, we will take ATG5 as the starting point to further study the molecular mechanism of PD pathogenesis, which may provide certain reference value for the diagnosis and treatment of neurodegenerative diseases related to ATG5 expression level to a certain extent.

## Data Availability

The data used to support the findings of this study are available from the corresponding author upon reasonable request.
